# Ogremorphin inhibits GPR68 mediated MUC5AC expression

**DOI:** 10.17912/micropub.biology.001639

**Published:** 2025-09-17

**Authors:** Leif R. Neitzel, Hussain F. Basrai, Charles C. Hong, Charles H. Williams

**Affiliations:** 1 Medicine, Michigan State University, East Lansing, Michigan, United States; 2 Henry Ford Health + Michigan State University Health Sciences, East Lansing, Michigan, United States

## Abstract

MUC5AC hypersecretion exacerbates airway obstruction in lung diseases, such as asthma, and promotes tumor growth. The expression of MUC5AC is driven by the pH-sensing receptor, GPR68. Herein we demonstrate that acidification (pH 6.4) increased MUC5AC mRNA and protein expression in a GPR68-dependent manner in A549 cells. Treatment with Ogremorphin (OGM), a specific GPR68 inhibitor, reduced MUC5AC mRNA and protein expression irrespective of pH. These findings were confirmed by shRNA-mediated knockdown of GPR68. These data suggest GPR68 inhibition by OGM as a potential therapy for mucus hypersecretion to improve airway clearance and reduce tumor progression in lung diseases.

**Figure 1. GPR68 regulates MUC5AC production:**
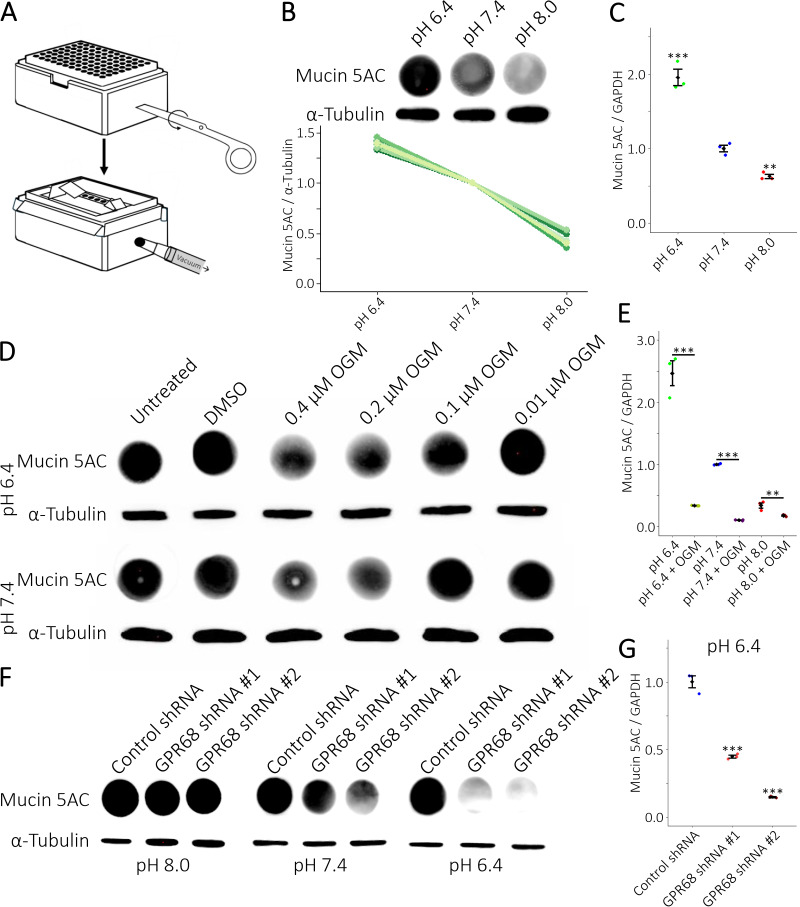
**(A)** Cartoon depicting the construction of a simple dot blot apparatus. **(B)** (Top) Dot blots of Mucin 5AC protein expression in A549 cells grown at different pHs overnight. (Bottom) Protein expression of n = 7 biological replicates normalized to pH 7.4 demonstrate reproducibility of the dot blots. **(C)** qRT-PCR analysis of Mucin 5AC expression shows a commensurate change in mRNA expression to protein expression in **(B)** . **(D)** Treatment of cells with Ogremorphin (OGM) decreases the expression of Mucin 5AC at both pH 6.4 and pH 7.4. **(E)** OGM decreases the expression of Mucin 5AC mRNA. **(F)** shRNA-mediated knockdown of GPR68 decreased Mucin 5AC protein expression at pH 7.4 and pH 6.4. **(G)** Knockdown of GPR68 with shRNAs at pH 6.4 reduced Mucin 5AC mRNA expression. **(B)** , **(D)** , and **(F)** Western blot analysis of α-tubulin shows equal loading of samples. **(C)** , **(E)** , and **(G)** The ratio of Mucin 5AC mRNA expression normalized to GAPDH mRNA as determined by qRT-PCR. **(D)** , and **(F)** representative examples from n ≥ 3 biological replicates. **(C)** , **(E)** , and **(G)** representative examples from n ≥ 3 biological replicates with n = 3 technical repeats. **(C)** and **(G)** mean +/- SD with significance determined by multiple two-tailed, equal variance, t-tests with Bonferroni Correction and an α-level of 0.01 at ** p<0.005 and 0.001 at ***p<0.0005. **(E)** mean +/- SD with significance determined by two-tailed, equal variance, t-tests with an α-level of 0.01 at ** p<0.01 and 0.001 at ***p<0.001 comparing DMSO control to 2 µM OGM treated sample. **(C)** and **(E)** normalized to expression at pH 7.4. **(G)** normalized to expression of control shRNA.

## Description

Mucin 5AC (MUC5AC) and Mucin 5B (MUC5B) are gel-forming mucins maintaining airway surface hydration and are the first line of innate airway defense. MUC5AC expression is low in healthy lungs but significantly upregulated in diseased lung states (Symmes et al., 2018). Conversely, MUC5B expression remains relatively stable or decreases in diseased lung states (Symmes et al., 2018). MUC5AC is critical for mucociliary clearance, clearing inhaled particles and pathogens, as well as clearing of recruited leukocytes, dead cells, and endogenous debris (Symmes et al., 2018). However, overproduction of MUC5AC worsens or results in disease states (Symmes et al., 2018). MUC5AC overproduction is a hallmark of respiratory diseases such as asthma, cystic fibrosis, chronic obstructive pulmonary disease, bronchiectasis, and lung cancer, as well as transient infectious or injurious responses (Krishn et al., 2018; Lakshmanan et al., 2016; Liu et al., 2013; Symmes et al., 2018).

Inflammation, infection, and tumorigenesis often acidify the microenvironment driving mucus hypersecretion (Jayaraman et al., 2001; Kodric et al., 2007; Liu et al., 2013; Ricciardolo et al., 2004; Sutoo et al., 2020; Szili et al., 2007). The G _q_ -coupled ovarian cancer G-protein-coupled receptor (OGR1 or GPR68) is activated by subtle extracellular acidification (nearly inactive at pH 7.4; fully active at pH 6.4) (Ludwig et al., 2003). GPR68 augments MUC5AC mRNA and protein expression in acidic conditions in human bronchial epithelial cells (Liu et al., 2013). We identified Ogremorphin (OGM), a specific small molecule inhibitor of GPR68 that induces ferroptosis and modulates radiosensitivity in cancer cells including the human lung adenocarcinoma A549 (Neitzel et al., 2024, 2025; Williams et al., 2024). A549 cells have been shown to endogenously express high levels of MUC5AC (Carterson et al., 2005; Lakshmanan et al., 2016). Furthermore, OGM attenuated *Staphylococcus aureus* -mediated lung injury, bacterial lipopolysaccharide-mediated endothelial dysfunction, and lung inflammation (Karki et al., 2024, 2025). Mounting evidence suggests microenvironmental acidification acts through GPR68 as a metabolic signal (reviewed in (Cornell et al., 2024)). Herein, we demonstrate that OGM attenuates acid-induced MUC5AC expression.

We first sought to determine if changes in pH alter MUC5AC expression in A549 cells. MUC5AC is a 641 kDa glycoprotein complicating western blot analysis. Therefore, we devised a simple dot blot apparatus to reliably quantify protein expression (Figure A, B). Western blot analysis was used to verify equal sample loading. At an acidic pH of 6.4, both MUC5AC protein and mRNA expression are upregulated (Figure B, C). This indicates that A549 cells express MUC5AC in a pH-dependent manner.

GPR68 regulates MUC5AC expression in human bronchial epithelial cells. We tested whether OGM, a GPR68 inhibitor, reduces the pH-dependent expression of MUC5AC in the human alveolar basal epithelial cell line, A549. At both pH 8.0 and pH 6.4 low concentrations of OGM inhibited MUC5AC protein production (Figure D). Concordantly, MUC5AC mRNA expression was reduced across all pHs (Figure E). At pH 6.4 MUC5AC mRNA expression was substantially lower than the DMSO control at pH 7.4. Using previously validated GPR68 shRNAs we knocked down the expression in A549 cells (Neitzel et al., 2025). MUC5AC protein expression decreased at pH 7.4 and 6.4 (Figure F). Furthermore, MUC5AC mRNA expression was reduced at pH 6.4. These data suggest chemical and genetic inhibition of GPR68 downregulates MUC5AC production in human lung cells irrespective of environmental pH. Our data confirms the findings in human bronchial epithelial cells and shows GPR68 inhibition by OGM as a potential treatment for lung diseases and cancer.

## Methods

**Dot blot apparatus**

A dot blot apparatus was constructed from a p20 pipette tip box (Fig. A). The cap was removed, and scissors were used to punch a rough small hole in the side of the box. Labeling tape sealed all top openings except sample holes plus one flanking each end. A filterless p20 pipette tip with the tip cut off, was inserted tightly into the side hole and connected to a vacuum via tubing. Methanol-soaked Polyvinylidene fluoride membranes (PVDF), cut with excess to optional be trimmed away post-blocking, were placed over the holes. The vacuum was adjusted until a slight divot was observed for each hole. 30 µl samples were added to the center of the divots and the vacuum was allowed to run until the sample had passed completely through the membrane. Membranes were then immediately placed in the blocking buffer.

**Dot and Western blots**

A549 cells were cultured in DMEM with 4.5g/L D-glucose, 25 mM HEPES, GlutaMAX supplement, 10% FBS, 100 units/mL of penicillin, and 100 µg/mL of streptomycin at 37°C and 5% CO _2_ . Cells were seeded on 100 mm plates in 10 mL of media overnight. The media was then removed, cells were washed once with DPBS and replaced with 30 mls fresh media at the desired pH. Media pH was adjusted with HCL or NaOH and filtered via 50 mL Steriflip. For OGM-treated samples, DMSO and/or OGM ensured equal DMSO volumes were added, and plates were briefly and gently swirled. Samples were then incubated for 24 hours. shRNA-treated samples were reverse transfected with Lipofectamine 3000, incubated for 24 hours, washed once with DPBS, and replaced with 30 mL of fresh media at the desired pH. Samples were then incubated for another 24 hours before collection in CelLytic M.

Protein aliquots (50 µg in 50 µl in CelLytic M) were prepared; 20 µl of the aliquot was added to 10 µls of Laemmli containing β-mercaptoethanol, both samples were boiled at 98°C for 10 mins, then cooled. The 30 µg sample was used for a dot blot. The 20 µg per 30 µl sample was run on a NuPage gel. Samples were transferred to a nitrocellulose membrane using a semidry apparatus. Dot blot and western membranes were blocked first in intercept blocking buffer (10 mins, RT) and then in 10% milk in PBS + 0.1% Tween 20 (PBST) for 30 mins. Dot blots were incubated with α-MUC5AC antibody (1:1000, 1 hour, RT with gentle rocking). Westerns were incubated in an α-α-tubulin antibody (1:1000, overnight, 4°C with gentle rocking). Membranes were then washed with copious amounts of PBST (5X 10 mins, with gentle rocking). They were then re-blocked (10% milk in PBST, 10 mins, with gentle rocking). HRP-Conjugated Secondary Antibody (1:1000, 1 hour, RT with gentle rocking). After washing with copious amounts of PBST (5X 10 mins, with gentle rocking) membranes were imaged on a chemidoc MP using chemiluminescence.

**qRT-PCR**

Samples were prepared as described above for blots. Post incubation in pH-adjusted media, samples were detached with the TrypLE express enzyme. Total RNA was then collected using an RNeasy mini kit and cDNA was generated. Samples were run with TaqMan probes on a QuantStudio 7. Graphs were created in R-studio, edited in Inkscape, and statistics were calculated with R 4.4.0.

## Reagents

**Table:** 


**Cell culture**			
**Reagent**	**Description**	**Catalogue #**	
A549 cells	Human lung cell carcinoma	ATCC	CCL-185
DMEM	High glucose, GlutaMAX supplement, HEPES	Gibco	10564011
FBS	Fetal bovine Serum	Gibco	A5670701
Penicillin-Streptomycin	10,000 U/mL	Gibco	15140122
TrypLE Express Enzyme	Cell dissociating reagent	Gibco	12604039
50 mL Steriflip	Filter with vacuum tube top	Sigma Aldrich	SCNY00020

**GPR68 inhibition**			
**Reagent**	**Description**	**Catalogue #**	
OGM	Ogremorphin resuspended in DMSO	-	-
hGPR68 shRNA #1	5’ GAGCTGTACCATCGACCATAC 3’	Vectorbuilder	VB221221-1235jft
hGPR68 shRNA #2	5’ CCACCGTTGTCACAGACAATG 3’	Vectorbuilder	VB221221-1234czj
Lipofectamine 3000	Transfection reagent	Invitrogen	L3000015

**Dot and Western blots**			
**Reagent**	**Description**	**Catalogue #**	
Pierce BCA protein Assay Kit	For determination of protein concentration	Thermo Scientific	23225
CelLytic M	Mammalian cell lysis reagent	Sigma Aldrich	C2978
4x Laemmli Sample Buffer	For preparation of SDS PAGE samples	Bio-Rad	161-0747
2-Mercaptoethanol	For preparation of SDS PAGE samples	Thermo Scientific Chemicals	125472500
PageRuler Plus	Pre-stained protein ladder	Thermo Scientific	26619
NuPAGE gels	Precast 4–12% gradient Bis-Tris protein gel	Invitrogen	NP0321BOX
Blot MES SDS running buffer	SDS PAGE running buffer	Invitrogen	B0002
Blot transfer buffer	SDS PAGE transfer buffer	Invitrogen	BT0006
Blotting Paper	Extra thick blot filter paper for SDS PAGE transfer	Bio-Rad	1703965
PVDF	Polyvinylidene fluoride membrane for dot blots	Bio-Rad	1620177
Nitrocellulose	Nitrocellulose membrane for western blots	Bio-Rad	1620115
Intercept blocking buffer	Membrane blocking buffer	LI COR	92770001
Milk	Blotting-grade nonfat dry milk	Bio-Rad	1706404
45M1	Mouse anti-Mucin 5AC antibody	Abcam	ab3649
DM1A	Mouse anti-alpha tubulin	Invitrogen	62204
Goat α-mouse GOXMO HRP high XADS	Goat anti-Mouse IgG (H+L) conjugated to HRP	Invitrogen	A16078
Chemi-luminescence substrate	SuperSignal West Pico PLUS for detection	Thermo Scientific	34580

**qRT-PCR**			
**Reagent**	**Description**	**Catalogue #**	
TaqMan Master Mix	TaqMan Universal Master Mix II, with UNG	Applied Biosystems	4440044
Hs01365616_m1	Probe for Mucin 5AC	Applied Biosystems	4331182
Hs02786624_g1	Probe for GAPDH	Applied Biosystems	4351182
RNeasy kit	RNeasy Mini Kit for collecting total RNA	Qiagen	74104
cDNA kit	High-capacity cDNA reverse transcription kit	Applied Biosystems	4374966

**Equipment**			
**Equipment**	**Description**	**Catalogue #**	
Accumet AB15 Basic	pH meter to preparing media	Fisher Scientific	-
XCell surelock mini-cell electrophoresis system	Protein gel electrophoresis system	Invitrogen	EI0001
Trans-Blot SD Semi-Dry Transfer Cell	Semi-Dry transfer system	Bio-Rad	1703940
ChemiDoc MP Imaging System	Membrane imaging system	Bio-Rad	12003154
GloMax-Multi+	For determination of protein concentrations	Promega	E9032
QuantStudio 7 Flex system	Quantification of qRT-PCR	Applied Biosystems	4485701
